# Edonerpic maleate prevents epileptic seizure during recovery from brain damage by balancing excitatory and inhibitory inputs

**DOI:** 10.3389/fncir.2024.1492043

**Published:** 2024-12-06

**Authors:** Yuki Katsuno, Susumu Jitsuki, Wataru Ota, Tomomi Yamanoue, Hiroki Abe, Takuya Takahashi

**Affiliations:** ^1^Department of Physiology, Yokohama City University Graduate School of Medicine, Yokohama, Japan; ^2^Department of Biochemistry, Mie University Graduate School of Medicine, Tsu, Japan

**Keywords:** AMPA receptors, edonerpic maleate, epileptic seizure, stroke, functional recovery, brain damage, synaptic plasticity

## Abstract

Functional recovery from brain damage, such as stroke, is a plastic process in the brain. The excitatory glutamate *α*-amino-3-hydroxy-5-methyl-4-isoxazole propionic acid receptor (AMPAR) plays a crucial role in neuronal functions, and the synaptic trafficking of AMPAR is a fundamental mechanism underlying synaptic plasticity. We recently identified a collapsin response mediator protein 2 (CRMP2)-binding compound, edonerpic maleate, which augments rehabilitative training-dependent functional recovery from brain damage by facilitating experience-driven synaptic delivery of AMPARs. In animals recovering from cryogenic brain injury, a potential compensatory area adjacent to the injured region was observed, where the injection of CNQX, an AMPAR antagonist, significantly attenuated functional recovery. In the compensatory brain area of animals recovering from cryogenic injury, the administration of edonerpic maleate enhanced both excitatory and inhibitory synaptic inputs at pyramidal neurons. In contrast, recovered animals that did not receive the drug exhibited augmentation of only excitatory synaptic input. The threshold of picrotoxin-induced epileptic seizure in recovered animals without edonerpic maleate treatment was lower than in intact animals and recovered animals with edonerpic maleate. Thus, edonerpic maleate enhances motor function recovery from brain damage by balancing excitatory and inhibitory synaptic inputs, which helps prevent epileptic seizures during recovery.

## Introduction

Brain damage such as stroke is a neurological condition that compromises a patient’s quality of life. The recovery from brain damage with rehabilitation is a plastic event in the central nervous system. At present, there are no effective pharmacological interventions to enhance the effects of rehabilitation, highlighting a critical need in current rehabilitation medicine. The manipulations of neural plasticity can be a promising approach for the pharmacological enhancement of rehabilitation. Synaptic trafficking of glutamate AMPA receptor (AMPAR) is a fundamental molecular mechanism underlying synaptic plasticity driven by experience (Nudo et al., 1996; Takahashi et al., 2003; Kessels and Malinow, 2009; Murphy and Corbett, 2009; Jitsuki et al., 2011; Murata et al., 2015; Miyazaki et al., 2020). We have recently identified a CRMP2-binding compound, edonerpic maleate, which accelerates motor function recovery after brain damage such as stroke by facilitating training-dependent synaptic trafficking of AMPAR in the compensatory brain regions. Administration of edonerpic maleate augments motor function recovery in non-human primates with internal capsule hemorrhage (Abe et al., 2018).

Post-stroke epilepsy compromises the rehabilitation process, and the prevention of it is a crucial clinical issue. We hypothesized that the functional compensation process can trigger epileptic malfunctions of the brain due to the elevation of the excitation of the compensatory brain regions (Nicolo et al., 2019). In this study, we located the compensatory brain region in the recovered animals with cryogenic brain injury, and AMPAR in this brain region mediates the recovery. AMPAR-mediated synaptic currents were increased in the compensatory brain region of naturally recovered animals, while inhibitory currents were unchanged in the region. In the compensatory brain regions of the recovered animals with edonerpic maleate administration, we observed increased amplitude of the miniature excitatory post-synaptic current (mEPSC) and miniature inhibitory post-synaptic current (mIPSC), balancing excitatory and inhibitory synaptic functions. The threshold to induce epileptic seizure with picrotoxin was lower in the naturally recovered animals than in control (sham-operated) animals and edonerpic maleate-induced recovered animals. Furthermore, blocking AMPAR in the compensatory brain region elevated the threshold to induce epileptic seizure. Thus, edonepric maleate can prevent post-stroke seizure by balancing excitatory and inhibitory synaptic functions.

## Materials and methods

### Animals

Male Long-Evans rats (280–300 g, 8 weeks of age) were purchased from Japan SLC. All animals were housed on a 12-h light/dark cycle with *ad libitum* access to water and food. All animal experiments were conducted in accordance with the Guide for the Care and Use of Laboratory Animals of Yokohama City University. The study protocol was approved by the Animal Care and Use Committee of Yokohama City University (approval number: F-A-22-053). All surgical procedures were performed under anesthesia, and every effort was made to minimize suffering.

### Reaching task

All behavioral experiments were performed under dim light conditions (20–25 lux) in a soundproof room. The rats were habituated to the room for 30 min prior to each behavioral session. The rats were food restricted (5 g/day/animal body weight) only 1 day before each session during the habituation, learning, and rehabilitative training periods, but water was provided *ad libitum*. The reaching task apparatus (width: 13.1 cm, height: 40 cm, and depth: 27 cm) was equipped with a 3-cm high food pellet stand. In addition, a 1.3 cm wide and 20 cm high slit was located at the bottom center or left or right of the apparatus (Xu et al., 2009). All rats were handled for 5 min each for 3 days. All rats were habituated to the apparatus for 4 days: the rats were allowed to move freely in the apparatus for 10 min, and pellets were placed on the food pellet stand. On day 4, the rats were evaluated to determine their dominant hand. The learning period was initiated after habituation was completed. During the learning period, the pellet was placed diagonally in front of the slit to force the rats to use their dominant forelimb in each session. Each session consisted of either 60 trials or 20 min for 5 days. Success was defined as the rats retrieving the pellet in a single-reach attempt without dropping it. Failure was defined by retrieving the pellet but dropping it, making many attempts to reach, flipping the pellet, or using the other (unaffected) forelimb. The success rate was calculated as the number of successful trials divided by the total number of trials. Only rats with a success rate of 40% or greater on the last day of learning were used in the experiment (Abe et al., 2018).

### Cryogenic injury

**Figure 1:**
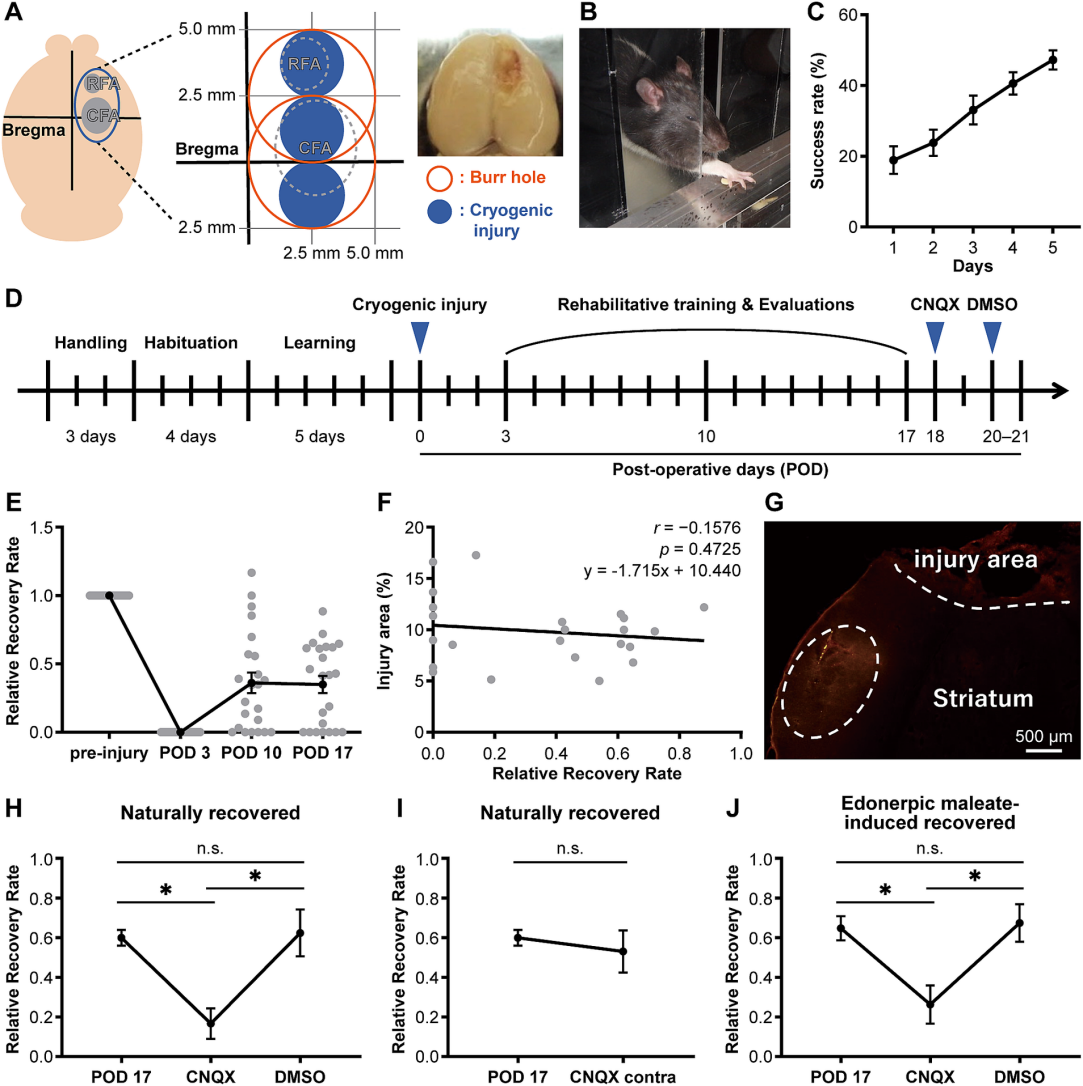
CNQX injection into the compensatory area attenuates functional recovery in both naturally recovered and edonerpic maleate-induced recovered rats. **(A)** (Left) Schematic of cortical cryogenic injury. CFA, Caudal forelimb area; RFA, Rostral forelimb area. (Middle) The coordinate of the cryogenic injury (mild injury for naturally recovered rats). (Right) Representative image of cryogenic injured rat brain. **(B)** Representative image of the reaching task in the rat. **(C)** Average success rate in the reaching task (*n* = 27). **(D)** Experimental design to evaluate forelimb motor function before/after cryogenic injury. CNQX, An AMPA receptor inhibitor; DMSO, Dimethyl sulfoxide as a control. **(E)** Time course of the average relative recovery rate (RRR) in naturally recovered rats following cryogenic injury (*n* = 23). **(F)** Correlation between injury area and RRR at postoperative day 17 (POD 17) (*n* = 23). Injury area (%) = (area of injury/area of whole brain) × 100. Data were analyzed using the Pearson correlation coefficient. **(G)** Representative image of cortical cryogenic injury area and CNQX-injected area (visualized by red fluorescence of co-injected Alexa 546). Scale bar: 500 μm. **(H)** CNQX injection into the compensatory area attenuates functional recovery in naturally recovered rats. *n* = 7 each. One-way ANOVA followed by Fisher’s LSD test (*p < 0.05). n.s., not significant. **(I)** CNQX injection into the contralateral side of the cryogenic injury did not affect functional recovery in naturally recovered rats. *n* = 7 each. Paired *t*-test. **(J)** CNQX injection into the compensatory area attenuates functional recovery in edonerpic maleate-induced recovered rats. *n* = 7 each. One-way ANOVA followed by Fisher’s LSD test (*p < 0.05).

After completing the motor learning phase of the reaching task, cryogenic injury (Tabuse et al., 2010) was introduced into the rat motor cortex using the Cryo system for ophthalmic surgery (Keeler Instruments). The rats were deeply anesthetized with 4% isoflurane/96% oxygen mixture. The skin overlying the skull was cut and gently pushed to the side. The rostral forelimb area (RFA) and the caudal forelimb area (CFA) were identified, and a region above the motor cortex (mild injury: 2.5 mm anterior to bregma, 2.5 mm lateral to bregma and 0 mm anterior to bregma, 2.5 mm lateral to bregma; severe injury: 2.5 mm anterior to bregma, 2.5 mm lateral to bregma) was gently pierced with a trephine (diameter, 5.0 mm) (Tennant et al., 2011; Starkey et al., 2012) (Figure 1A). A metal probe chilled with CO_2_ gas was applied to the burr hole in cycles consisting of a 30-s application, two cycles for each of the three points for mild injury, or four cycles for each of the two points for severe injury employed for each rat. After surgery, the skin was sutured. The rats were kept on a heating pad during the procedures and returned to their home cages after regaining mobility. Animals with a 45% or greater decrease in success rate compared to pre-injury (last day of learning) were defined as cryogenically injured.

### Rehabilitative training

During the period of rehabilitative training, the rats were placed in the apparatus for 30 min with some pellets on the stand in front of the slit so that they could reach the pellets by their affected forelimb. Edonerpic maleate (84 mg/kg body weight) or vehicle (distilled water) was administered 30 min before training (Abe et al., 2018).

### Evaluation of motor functions

The relative recovery rate (RRR) in the reaching task was used to evaluate forelimb motor function. The RRR was calculated as follows:


$$\begin{array}{l} RRR=\left(\begin{array}{l} \mathrm{success rate}\;\mathrm{at}\;\mathrm{time of evaluation}\;\left[\mathrm{POD}\;3,10,17\mathrm{,etc}.\right]\\ \;-\mathrm{success rate}\;\mathrm{at}\;\mathrm{post-injury evaluation}\;\left[\mathrm{POD}\;3\right] \end{array}\right)/\\ \left(\begin{array}{l} \mathrm{success rate}\;\mathrm{at}\;\mathrm{last}\;\mathrm{day}\;\mathrm{of learning}\;[\mathrm{pre-injury]}\\ \;-\mathrm{success rate}\;\mathrm{at}\;\mathrm{post-injury evaluation}\;\left[\mathrm{POD}\;3\right] \end{array}\right) \end{array}$$

**Figure 2:**
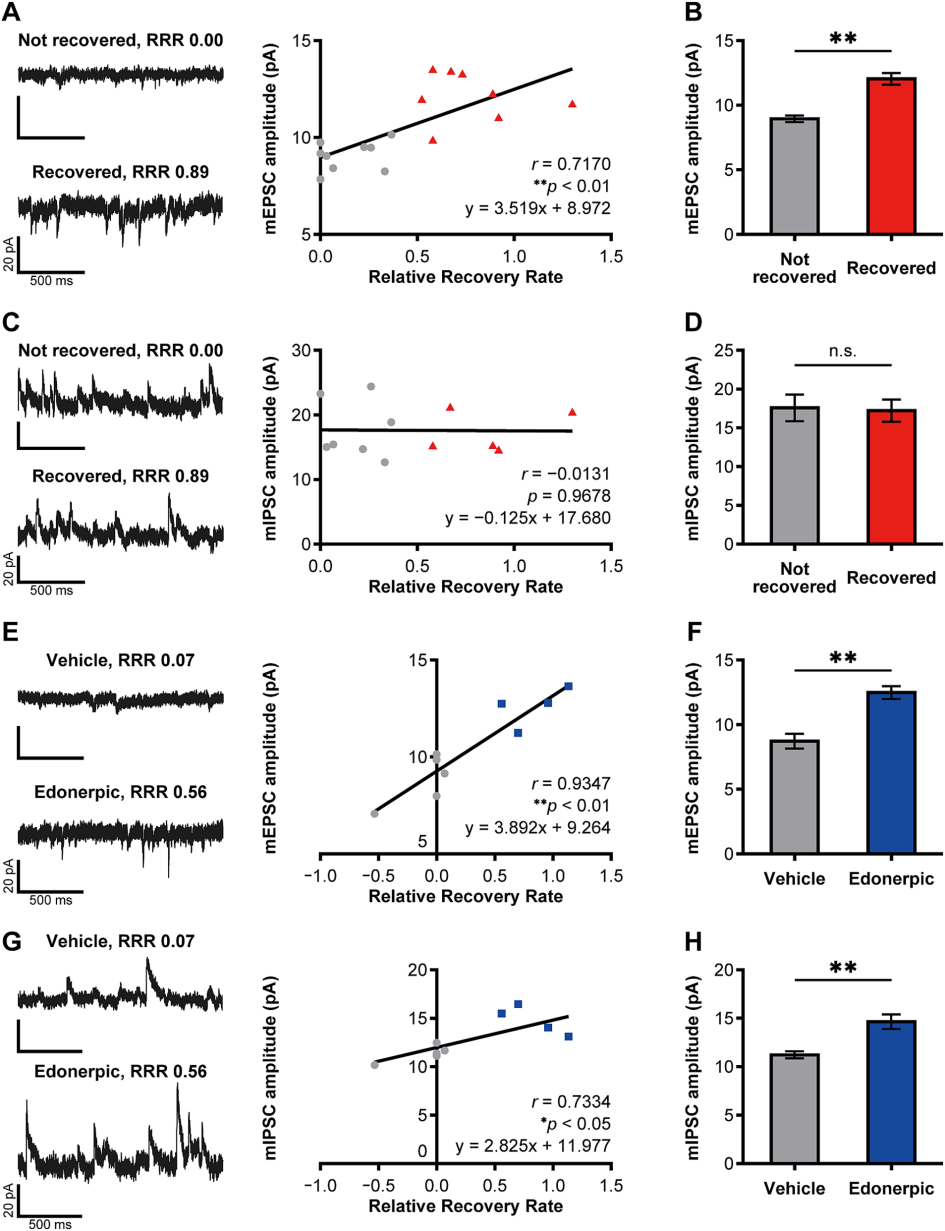
Edonerpic maleate balances excitatory/inhibitory synaptic inputs onto pyramidal neurons in the compensatory cortical region during functional recovery after brain injury. **(A)** (Top left) Representative spontaneous mEPSC from layer 5 pyramidal neurons in the compensatory area after 2 weeks of rehabilitative training in mildly injured but “not recovered” rats [Relative Recovery Rate (RRR): 0.00]. (Bottom left) Representative spontaneous mEPSC from layer 5 pyramidal neurons in the compensatory area following 2 weeks of rehabilitative training in mildly injured “naturally recovered” rats (RRR: 0.89). (Right) Correlation between mEPSC amplitudes (pA) and RRR in mildly injured rats. The gray circles indicate the plot of “not recovered (RRR < 0.4)” rats, and the red triangles indicate the plot of “recovered (RRR ≥ 0.4)” rats (same in **C**). Data were analyzed using the Pearson correlation coefficient. **(B)** Average amplitudes of spontaneous mEPSCs. Comparison of not recovered (*n* = 9 animals, 11 cells) and recovered (*n* = 8 animals, 14 cells) rats. Unpaired *t*-test (**p < 0.01). **(C)** (Top left) Representative spontaneous mIPSC from layer 5 pyramidal neurons in the compensatory area after 2 weeks of rehabilitative training in mildly injured but “not recovered” rats (RRR: 0.00). (Bottom left) Representative spontaneous mIPSC from layer 5 pyramidal neurons in the compensatory area after 2 weeks of rehabilitative training in mildly injured “naturally recovered” rats (RRR: 0.89). (Right) Correlation between mIPSC amplitudes (pA) and RRR in mildly injured rats. Data were analyzed using the Pearson correlation coefficient. **(D)** Average amplitudes of spontaneous mIPSCs. Comparison of not recovered (*n* = 7 animals, seven cells) and recovered (*n* = 5 animals, eight cells) rats. Unpaired *t*-test. n.s., not significant. **(E)** (Top left) Representative spontaneous mEPSC from layer 2/3 pyramidal neurons in the compensatory area after 2 weeks of rehabilitative training in control (severely injured, vehicle-treated) rats (RRR: 0.07). (Bottom left) Representative spontaneous mEPSC from layer 2/3 pyramidal neurons in the compensatory area after 2 weeks of rehabilitative training with edonerpic maleate-induced recovered rats (severely injured, edonerpic maleate-treated) (RRR: 0.56). (Right) Correlation between mEPSC amplitudes (pA) and RRR in severely injured rats. The gray circles indicate the plot of “vehicle-treated control (RRR < 0.4)” rats, and the blue squares indicate the plot of “edonerpic maleate-induced recovered (RRR ≥ 0.4)” rats (same in **G**). Data were analyzed using the Pearson correlation coefficient. **(F)** Average amplitudes of spontaneous mEPSCs. Comparison of vehicle-treated control (*n* = 5 animals, 12 cells) and edonerpic maleate-induced recovered (*n* = 4 animals, nine cells) rats. Unpaired *t*-test (**p < 0.01). **(G)** (Top left) Representative spontaneous mIPSC from layer 2/3 pyramidal neurons in the compensatory area after 2 weeks of rehabilitative training in control rats (RRR: 0.07). (Bottom left) Representative spontaneous mIPSC from layer 2/3 pyramidal neurons in the compensatory area after 2 weeks of rehabilitative training with edonerpic maleate-induced recovered rats (RRR: 0.56). (Right) Correlation between mIPSC amplitudes (pA) and RRR in severely injured rats. Data were analyzed using the Pearson correlation coefficient. **(H)** Average amplitudes of spontaneous mIPSCs. Comparison of control (*n* = 5 animals, 12 cells) and edonerpic maleate-induced recovered (*n* = 4 animals, nine cells) rats. Unpaired *t*-test (**p < 0.01).

**Figure 3:**
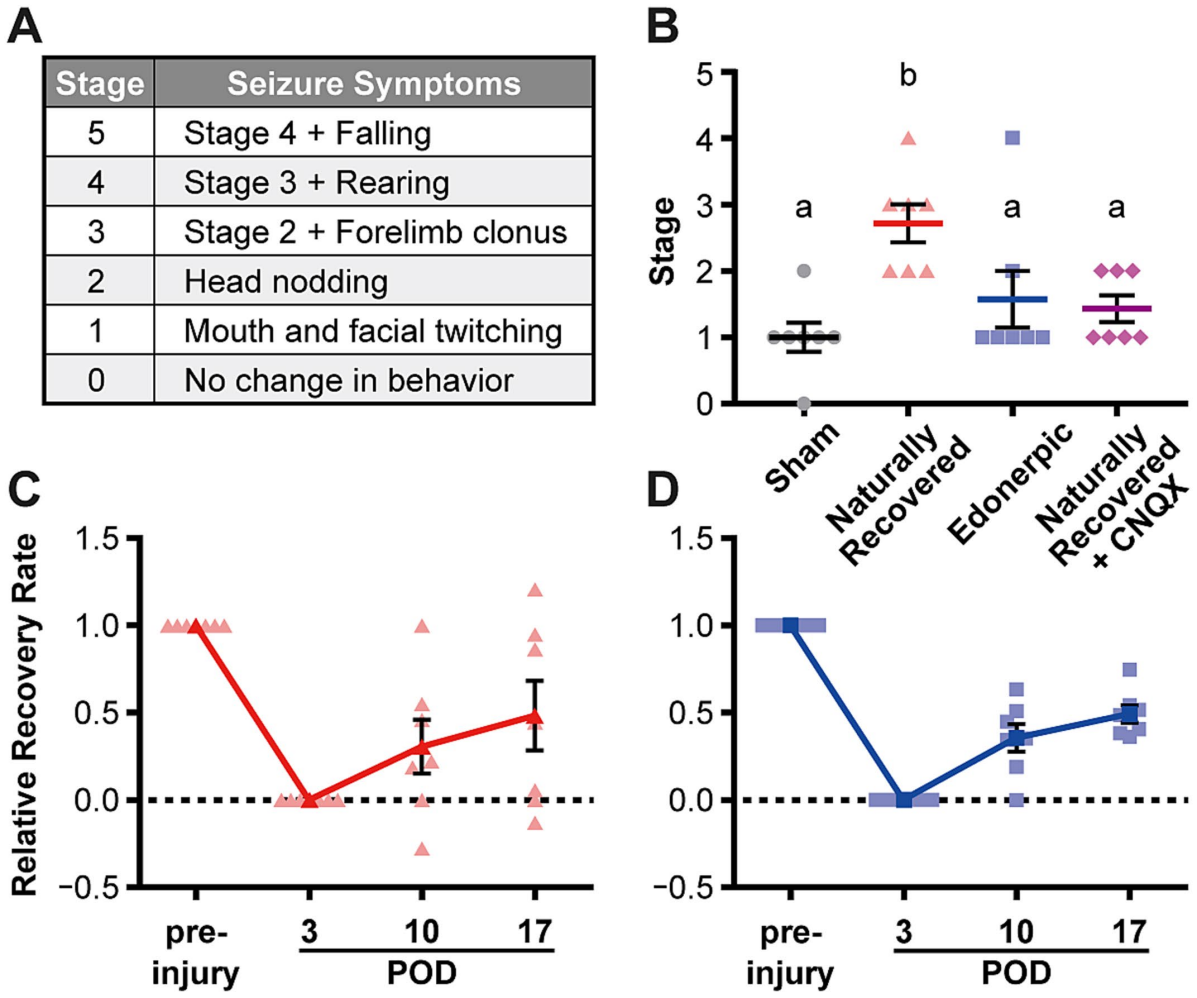
Edonerpic maleate prevents epileptic seizure during recovery from brain damage, and CNQX inhibition of AMPA receptor function in the compensatory region of naturally recovered animals downregulated sensitivity to picrotoxin-induced epilepsy. **(A)** Racine classification used to evaluate the stage of epilepsy (Racine, 1972; Cela et al., 2019). **(B)** Stage of epilepsy in picrotoxin-treated animals [sham-operated, naturally recovered (rehabilitative training only), edonerpic maleate-induced recovered (edonerpic maleate administration + rehabilitative training), and naturally recovered + CNQX-injected rats] at POD 17. *n* = 7 each. One-way ANOVA followed by Fisher’s LSD test. The different letters indicate significant differences between groups (*p* < 0.05). **(C)** Time course of the average RRR in naturally recovered rats following cryogenic injury. **(D)** Time course of the average RRR in edonerpic maleate-induced recovered rats following cryogenic injury.

a) Naturally recovered rats (Figures 1A–I, 2A–D, 3B,C): After the learning period (i.e., before the rehabilitative training), rats were induced with a cryogenic injury (mild injury) and were tested in the reaching task on postoperative days (PODs) 3, 10, and 17. In this mild injury model, approximately 60% of the animals recovered forelimb function with rehabilitative training.
b) Edonerpic maleate-treated rats (Figures 1B–D, J, 2E–H, 3B, D): After the learning period, rats were induced with a cryogenic injury (severe injury) and tested in the reaching task on PODs 3, 10, and 17. In this severe injury model, the rats were unable to recover forelimb function without the administration of edonerpic maleate along with rehabilitative training. The rats were tested 30 min after receiving either edonerpic maleate (84 mg/kg body weight) or vehicle administration.

### Electrophysiology

Electrophysiological analysis was performed according to previous studies (Jitsuki et al., 2011; Abe et al., 2018). After the final assessment of forelimb motor function, rats were anesthetized with an isoflurane/oxygen mixture, and the brains were removed and quickly transferred to gassed (95% O_2_ and 5% CO_2_) ice-cold dissection buffer. Coronal brain slices were cut (400 μm, Leica VT1200) in the dissection buffer. The slices were then incubated in gassed artificial cerebrospinal fluid (ACSF). Patch recording pipettes (3–7 MΩ) were filled with intracellular solution as described previously (Jitsuki et al., 2011; Miyazaki et al., 2012; Nakajima et al., 2016). For the recording of mEPSCs and mIPSCs in the layer 5 or 2/3 pyramidal neurons of the peri-injured cortex, sections corresponding to Figures 11–14 in The Rat Brain in Stereotaxic Coordinates (compact sixth edition) (Paxinos and Watson, 2009) were selected. The recording chamber was perfused with 0.5 μM tetrodotoxin (TTX)-containing ACSF as described previously (Mitsushima et al., 2013). The detection threshold for mEPSCs was set at 2 × RMS noise. The mEPSCs and mIPSCs were basically obtained from the same neurons using reversal potentials, but some neurons only recorded mEPSCs due to time constraints. The E/I ratio was calculated by dividing the mEPSC value of each recorded cell by the mIPSC value.

### Experimental inhibition of compensatory field function using AMPA receptor inhibitor

After 14 days of rehabilitative training (POD 17), rats with an RRR greater than 0.40 by motor function assessment were used in this experiment. A mixture of 0.2 μL of 40 μM 6-cyano-7-nitroquinoxaline-2,3-dione (CNQX), an AMPA receptor inhibitor, and a fluorescent dye marker (Alexa 546) was injected locally into the cortex of the injured side (compensatory field) on POD 18, and motor function was assessed after 30 min. After 2–3 days (POD 20–21), 0.2 μL of 0.2% dimethyl sulfoxide (DMSO) was injected locally at the same site in the same rats as the control, and motor function was assessed after 30 min. The compensatory field in this study corresponds to the primary somatosensory cortex, jaw region (S1J) in normal animals. There were nine injection sites: the tip of a glass needle inclined at 30 degrees to the horizontal plane and positioned 5.0 mm laterally (on the injured side) from the bregma, three sites on the anterior side (1.1 mm, 1.6 mm, and 2.1 mm), and three sites 1.5 mm, 2.5 mm, and 3.5 mm further from each site in the direction of inclination. The same injections were made on the contralateral side of the injury as a control.

### Jugular vein catheterization for drug administration

A medical tube (Kaneka Medix Corporation)^1^ with an inner diameter of 0.3 mm and an outer diameter of 0.64 mm was cut into 12.5 cm lengths, and a 16-cm silk suture (Natsume Seisakusho Co., Ltd.)^2^ was tied orthogonally to the tube at a position 2.5 cm from the tip and sticked with adhesive. Rats were anesthetized with an isoflurane/oxygen mixture, and the jugular vein near the atrium was exposed. After penetrating the jugular vein with a needle attached to a catheter, the catheter was removed from the needle, and the 2.5-cm tip of the catheter was inserted into the jugular vein (Furuta et al., 2001). To prevent blood coagulation in the catheter, 1% heparinized saline was administered. After assuring reverse blood flow and negative pressure on the catheter, a silk suture was connected to the muscle near the jugular vein. The other tip of the catheter was inserted outside the body, through the dorsal subcutaneous area. To prevent blood from refluxing into the catheter, a plug was placed at the tip of the catheter. Following surgery, the skin was sutured. The rats were kept on a heating pad during the procedures and then returned to their home cages after they regained mobility.

### Epileptic induction using picrotoxin

Chemical induction of epileptic seizures was performed using picrotoxin, a *γ*-aminobutyric acid type A receptor (GABA_A_Rs) antagonist. Picrotoxin (0.5 mg/kg body weight) was administered via an implanted jugular vein cannula on POD 17 to naturally recovered rats, edornerpic maleate-treated rats, and control (sham-operated) rats. The level of epileptic seizures induced was then observed (Pericić and Bujas, 1997). The Racine classification (Racine, 1972; Cela et al., 2019) was used to determine the stage of epilepsy. Similar studies were conducted in naturally recovered rats with and without CNQX suppression of AMPA receptor function in the compensatory cortical region.

### Statistics and graphs

The Mann–Whitney *U* test or Student’s *t*-test was used to compare two independent groups. One-way ANOVA followed by Fisher’s LSD test was used to compare more than three independent groups. Pearson correlation analysis was performed to determine the relationship between the two variables. *p* values <0.05 were considered statistically significant. Statistical analyses were conducted using GraphPad Prism 8 (Graph Pad Software). The error bars on the graphs indicate the standard error of the mean (SEM).

## Results

### Characterization of the compensatory cortical region of recovered animals with cryogenic injury

In our previous study, we found that edonerpic maleate enhanced motor function recovery from brain damage caused by cryogenic injury in mice. We found that the compensatory brain region was located adjacent to the injured area in recovered mice (Abe et al., 2018). In this study, we first examined the compensatory brain regions in rats following cryogenic injury to the motor cortex (Figure 1A). We induced cryogenic injury in the motor cortex of rats that had been trained to reach for food pellets with their forelimbs (Figures 1A–C). Forelimb reaching movements were analyzed based on the success rate of retrieving the food pellets (Figure 1C). Following the introduction of cryogenic injury, we conducted rehabilitative training for injured animals to retrieve food pellets (Figure 1D). Some animals showed moderate recovery in success rate after 2 weeks of forelimb rehabilitation for retrieving food pellets, while we observed variations in recovery among animals (Figure 1E), which did not depend on the volume of injury in our experimental system (Figure 1F). Based on our previous study, which showed that the compensatory brain region in recovered animals administered with edonerpic maleate was located adjacent to the injured region (Abe et al., 2018), we hypothesized that the compensatory brain region would also be adjacent to the injured region of rats with cortical cryogenic injury. We administered CNQX (6-cyano-7-nitroquinoxaline-2,3-dione), an antagonist of AMPAR, into the brain regions adjacent to the injured area of recovered animals without edonerpic maleate (Figure 1G). The local injection of CNQX in this area attenuated recovered forelimb function, indicating that it was a compensatory brain region (Figures 1G,H). Furthermore, 2–3 days after the injection of CNQX, which attenuated recovered forelimb function, the animals regained functional recovery to the equivalent level as we observed before the injection, which was not disrupted by an additional injection of DMSO into the same brain region as the CNQX injection (Figures 1D,H). These results indicated that functional recovery from brain damage requires AMPAR-mediated synaptic function in the compensatory brain regions. When we injected CNQX into the contralateral hemisphere, recovered forelimb motor function was maintained, further proving that the CNQX injection area adjacent to the injured region was the compensatory brain region of the recovery (Figure 1I). To identify the compensatory brain regions of edonerpic maleate-induced recovered rats, we used severe cryogenic injury that prevented rats from recovering without edonerpic maleate (Supplementary Figure 1). We administered CNQX into brain regions adjacent to the cryogenic injury area of rats treated with edonerpic maleate. This procedure aggravated previously recovered forelimb motor function, whereas the injection of DMSO in the same area did not affect recovered forelimb function (Figure 1J), indicating that the region adjacent to the injury site is a compensatory brain region where AMPAR mediates functional recovery.

### Analysis of the microcircuits in the compensatory cortical region of recovered animals with cryogenic injury

We further investigated how edonerpic maleate-induced increase of synaptic plasticity alters microcircuits in the cortical regions responsible for the functional compensation following brain damage. We prepared acute cortical slices and performed whole cell recordings from layer 5 pyramidal neurons in the abovementioned potential functional compensatory cortical region (Figures 2A–D). We detected a significant positive correlation between the amplitude of mEPSCs and the functional recovery rate in rats without the administration of edonerpic maleate (Figure 2A). Consistent with this finding, we found an increased amplitude of mEPSCs in these brain regions of recovered animals compared to non-recovered rats (Figure 2B). We also detected the difference in the frequency of mEPSCs of these neurons between recovered and non-recovered rats (Supplementary Figures 2A,B). We did not observe a correlation between the amplitude of mIPSCs and functional recovery in these pyramidal neurons (Figure 2C). Additionally, no differences were detected in the amplitude or frequency of mIPSCs between recovered and non-recovered animals (Figure 2D; Supplementary Figures 2C,D).

We next investigated the alterations of microcircuits within the compensatory brain regions of rats with cryogenic injury after administering edonerpic maleate. We introduced more severe cryogenic injury than in the previously mentioned animals, resulting in no recovery for vehicle-treated injured rats. In contrast, administration of edonerpic maleate led to significant recovery, as previously reported in both mice and non-human primates (Abe et al., 2018) (Supplementary Figure 1). We prepared acute cortical slices and performed whole-cell recordings from layer 2/3 pyramidal neurons in the compensatory cortical brain regions identified in the abovementioned experiments (Figures 2E–H). We detected a significant positive correlation between the amplitude and frequency of mEPSCs and the functional recovery rate (Figure 2E; Supplementary Figure 2E). Additionally, the amplitude of mEPSCs in drug-treated recovered animals was greater than that in vehicle-treated non-recovered animals (Figure 2F). The frequency of mEPSCs was unchanged between these two groups (Supplementary Figure 2F). Interestingly, we also detected a positive correlation between the amplitude of mIPSCs and the functional recovery rate (Figure 2G). Furthermore, we observed an increased amplitude of mIPSC at pyramidal neurons in layer 2/3 of the compensatory brain regions of edonerpic maleate-treated recovered animals than those of vehicle-treated non-recovered animals (Figure 2H), while the frequency was unaltered in these animals (Supplementary Figures 2G,H). The calculated E/I ratio of post-synaptic current amplitude and frequency was significantly increased in naturally recovered rats (Supplementary Figures 3A,B), but remained unchanged in edonerpic maleate-induced recovered rats (Supplementary Figures 3C,D). These results indicate that edonerpic maleate balances excitatory/inhibitory synaptic inputs onto pyramidal neurons in the compensatory cortical region by facilitating synaptic trafficking of AMPAR and GABAR during functional recovery following brain injury.

### Edonerpic maleate prevents epileptic seizures during recovery from brain damage

Post-stroke epileptic seizure hampers recovery through rehabilitation. As previously described, recovered animals from cortical cryogenic injury without drug treatment exhibited an increase in the amplitude of mEPSCs but not mIPSCs in the pyramidal neurons of layer 5 in the compensatory brain region (Figures 2A–D). In contrast, animals administered edonerpic maleate showed increases in both mEPSCs and mIPSCs in these neurons (Figures 2E–H). Thus, we hypothesize that the increased excitability of the compensatory region in recovered animals without drugs can lower the threshold for inducing epilepsy during the recovery process. We induced epilepsy in control (sham-operated) animals or the animals naturally recovered from cortical cryogenic injury without edonerpic maleate by injecting picrotoxin. We found that naturally recovered animals without the drug treatment exhibited a higher sensitivity to induce epileptic seizure in response to the injection of picrotoxin than sham-operated animals (Figures 3A–C). Layer 2/3 pyramidal neurons in the compensatory brain regions of recovered animals treated with edonerpic maleate exhibited increases in the amplitude of both mEPSCs and mIPSCs (Figures 2E–H). Thus, we hypothesized that these recovered animals are resistant to epileptogenesis when treated with edonerpic maleate. Consistent with this hypothesis, the seizure grade of these animals injected with picrotoxin was lower than those without edonerpic maleate and comparable to that of intact animals (Figures 3A–D). To examine if the elevated excitability by the increased AMPAR-mediated synaptic currents in the compensatory region of the recovered animals without the administration of edonerpic maleate is responsible for epileptogenesis, we injected CNQX (6-cyano-7-nitroquinoxaline-2,3-dione), a competitive antagonist of AMPA/Kainate receptors, into the compensatory region of recovered animals from cryogenic injury of the motor cortex without edonerpic maleate and induced epileptic seizure by the injection of picrotoxin. The injection of CNQX downregulated the sensitivity to induce epilepsy by picrotoxin (Figure 3B), indicating that increased AMPAR-mediated synaptic currents and unchanged inhibitory synaptic inputs in the compensatory brain region were responsible for the epileptogenesis. These results suggested that the administration of edonerpic maleate not only facilitates motor functional recovery of animals with brain injury but also prevents epileptic seizure during recovery.

## Discussion

Epileptic seizure during the recovery process from brain damage such as stroke is a serious aversive event and often hampers the recovery process. We have previously demonstrated that AMPAR is trafficked into synapses of compensatory cortical regions during recovery and that this process is required for functional recovery (Jitsuki et al., 2011; Abe et al., 2018). It is possible that synaptic AMPAR trafficking elevates excitability in the compensatory brain regions that can potentially cause epileptic seizures during recovery after brain damage. In this study, we demonstrated that the compensatory brain region of recovered animals from cryogenic injury of the motor cortex without edonerpic maleate exhibited increased AMPAR-mediated synaptic currents but not GABAR-mediated synaptic currents and was responsible for lowering the threshold to induce an epileptic seizure in response to the injection of picrotoxin (Figures 2B,D, 3B). It remains to be elucidated how the compensatory brain area turns from “functional” to “epileptic.” In contrast, edonerpic maleate-treated injured animals were less sensitive to inducing an epileptic seizure in response to picrotoxin (Figure 3B). Given that edonerpic maleate-treated recovered animals from cryogenic injury showed increased amplitudes of mEPSCs and mIPSCs (Figures 2F,H), potential downregulation of GABAR-mediated currents in the compensatory region could change the compensational brain region from “functional” to “epileptic.” The mechanisms underlying the potential downregulation of inhibitory synaptic function in the “epileptic” compensatory brain regions require further investigation.

In this study, we used two different levels of injury: one is “mild injury” to mimic and validate the changes in the brain during the spontaneous recovery process (i.e., naturally recovered model), and the other is “severe injury” to examine the effects of edonerpic maleate. As described in the Materials and Methods section, these severely injured rats are unable to recover forelimb function without edonerpic maleate administration and rehabilitative training. As a technical limitation, the layer recording mEPSCs/IPSCs differed between the mild injury model (layer 5) and the severe injury model (layer 2/3). It was desirable to perform the experiments in the same layer, but unfortunately, it was technically impossible to record mEPSCs/IPSCs from layer 5 of edonerpic maleate (or vehicle) treated rats due to their severe injury. However, it is worth noting that layer 2/3 and layer 5 pyramidal neurons have been reported to show similar morphological changes (loss of dendritic spines) following focal stroke (Brown et al., 2008).

Our results suggest a protective effect of edonerpic maleate in the prevention of epileptic seizures during recovery from brain damage. Edonerpic maleate treatment can enhance not only AMPAR-but also GABAR-mediated currents in the compensatory brain regions (Figures 2F,H). This shows that edonerpic maleate affects common molecules or pathways that regulate AMPAR and GABAR trafficking, or that it regulates them independently. Although AMPARs and GABARs have distinct functions in neuronal excitability, their dynamics in neurons are remarkably similar (Li et al., 2022). Both receptors are synthesized in the endoplasmic reticulum (ER) (Greger et al., 2002; Saliba et al., 2008). After assembly in the Golgi apparatus, both receptors are transported to the synapse via vesicles, which are frequently mediated by motor proteins such as myosin along actin and kinesin and dynein along microtubules (Broutman and Baudry, 2001; Malinow and Malenka, 2002; Jacob et al., 2008; Schlager and Hoogenraad, 2009). Internalization of both AMPARs and GABARs into the cytoplasm is mediated by clathrin (Carroll et al., 2001; Kanematsu et al., 2007), and recycling of both AMPARs and GABARs is regulated by kinesin superfamily proteins (KIFs) (Wang and Xu, 2015; Li et al., 2022). Edonerpic maleate is a compound that binds to CRMP2, and CRMP2 mediates edonerpic-induced functional recovery (i.e., trafficking of AMPARs into the synapse) via actin depolymerizing factor (ADF)/cofilin activation (Abe et al., 2018). Given its function as an enhancer of neuronal plasticity, it is reasonable that GABARs would also be affected by edonerpic maleate. The key target molecules/pathways of edonerpic maleate that upregulate GABARs should be identified in future research.

Taken together, edonerpic maleate, a potential accelerator of functional recovery after stroke, can prevent post-stroke epileptogenesis by balancing the excitatory and inhibitory synaptic functions.

## Data Availability

The raw data supporting the conclusions of this article will be made available by the authors, without undue reservation.

## Footnotes

^1^https://www.kaneka-med.jp/english/

^2^https://www.nazme.co.jp/english/

## Supplementary material

SUPPLEMENTARY FIGURE 1 Forelimb motor function is restored only in the edonerpic maleate treated group in cases of severe cortical cryogenic injury. Time course of the average RRR after severe cortical cryogenic injury and rehabilitation in rats treated with Edonerpic maleate (*n* = 9) or Vehicle (*n* = 6). Unpaired *t*-test between groups in the same POD (**p* < 0.05).

SUPPLEMENTARY FIGURE 2 The balance of spontaneous mEPSC/IPSC frequency is also disrupted in naturally recovered rats and maintained in edonerpic maleate-induced recovered rats. **(A)** Correlation between mEPSC frequency (Hz) and RRR in mildly injured rats. Gray circles indicate the plot of “not recovered (RRR < 0.4)” rats, and red triangles indicate the plot of “recovered (RRR ≥ 0.4)” rats (same in **C**). Data were analyzed by Pearson correlation coefficient. **(B)** Average frequencies of spontaneous mEPSCs. Comparison of not recovered (*n* = 9 animals, 11 cells) and recovered (*n* = 8 animals, 14 cells) rats. Unpaired *t*-test (***p* < 0.01). **(C)** Correlation between mIPSC frequency (Hz) and RRR in mildly injured rats. Data were analyzed by Pearson correlation coefficient. **(D)** Average frequencies of spontaneous mIPSCs. Comparison of not recovered (*n* = 7 animals, seven cells) and recovered (*n* = 5 animals, eight cells) rats. Unpaired *t*-test. n.s., not significant. **(E)** Correlation between mEPSC frequency (Hz) and RRR in severely injured rats. Gray circles indicate the plot of “vehicle treated control (RRR < 0.4)” rats, and blue squares indicate the plot of “edonerpic maleate-induced recovered (RRR ≥ 0.4)” rats (same in **G**). Data were analyzed by Pearson correlation coefficient. **(F)** Average frequencies of spontaneous mEPSCs. Comparison of vehicle treated control (*n* = 5 animals, 12 cells) and edonerpic maleate-induced recovered (*n* = 4 animals, nine cells) rats. Unpaired *t*-test. n.s., not significant. **(G)** Correlation between mIPSC frequency (Hz) and RRR in severely injured rats. Data were analyzed by Pearson correlation coefficient. **(H)** Average frequencies of spontaneous mIPSCs. Comparison of control (*n* = 5 animals, 12 cells) and edonerpic maleate-induced recovered (*n* = 4 animals, nine cells) rats. Unpaired *t*-test. n.s., not significant.

SUPPLEMENTARY FIGURE 3 Disrupted E/I ratio in naturally recovered rats, but not in edonerpic maleate-induced recovered rats. **(A)** E/I ratio of post-synaptic current amplitude in mildly injured rats. Comparison of not recovered (*n* = 7 animals, seven cells) and recovered (*n* = 5 animals, eight cells) rats. Unpaired *t*-test (**p* < 0.05). **(B)** E/I ratio of post-synaptic current frequency in mildly injured rats. Comparison of not recovered (*n* = 7 animals, seven cells) and recovered (*n* = 5 animals, eight cells) rats. Unpaired *t*-test (**p* < 0.05). **(C)** E/I ratio of post-synaptic current amplitude in severely injured rats. Comparison of vehicle treated control (*n* = 5 animals, 12 cells) and edonerpic maleate-induced recovered (*n* = 4 animals, nine cells) rats. Unpaired *t*-test. n.s., not significant. **(D)** E/I ratio of post-synaptic current frequency in severely injured rats. Comparison of vehicle treated control (*n* = 5 animals, 12 cells) and edonerpic maleate-induced recovered (*n* = 4 animals, nine cells) rats. Unpaired *t*-test. n.s., not significant.
