# Supplementary figures and images for: Edonerpic maleate prevents epileptic seizure during recovery from brain damage by balancing excitatory and inhibitory inputs
Source: Front Neural Circuits. 2024 Dec 6;18:1492043. doi: 10.3389/fncir.2024.1492043 (PMC11660091; doi:10.3389/fncir.2024.1492043)

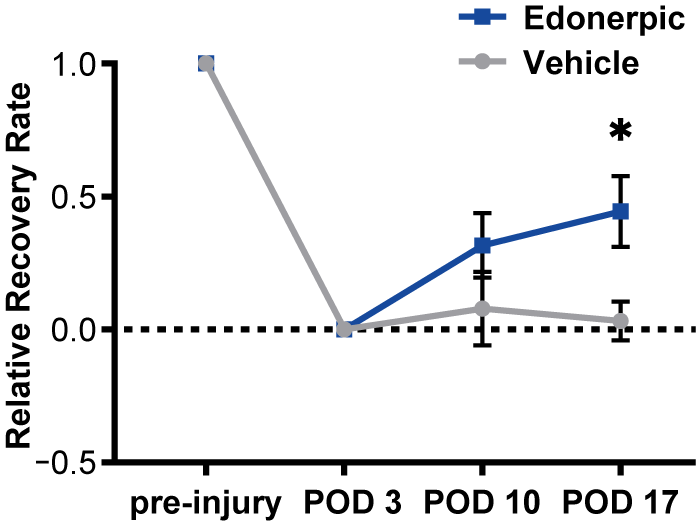

Supplement: SUPPLEMENTARY FIGURE 1 — Forelimb motor function is restored only in the edonerpic maleate treated group in cases of severe cortical cryogenic injury. Time course of the average RRR after severe cortical cryogenic injury and rehabilitation in rats treated with Edonerpic maleate (n = 9) or Vehicle (n = 6). Unpaired t-test between groups in the same POD (*p < 0.05). [file Image_1.TIF]

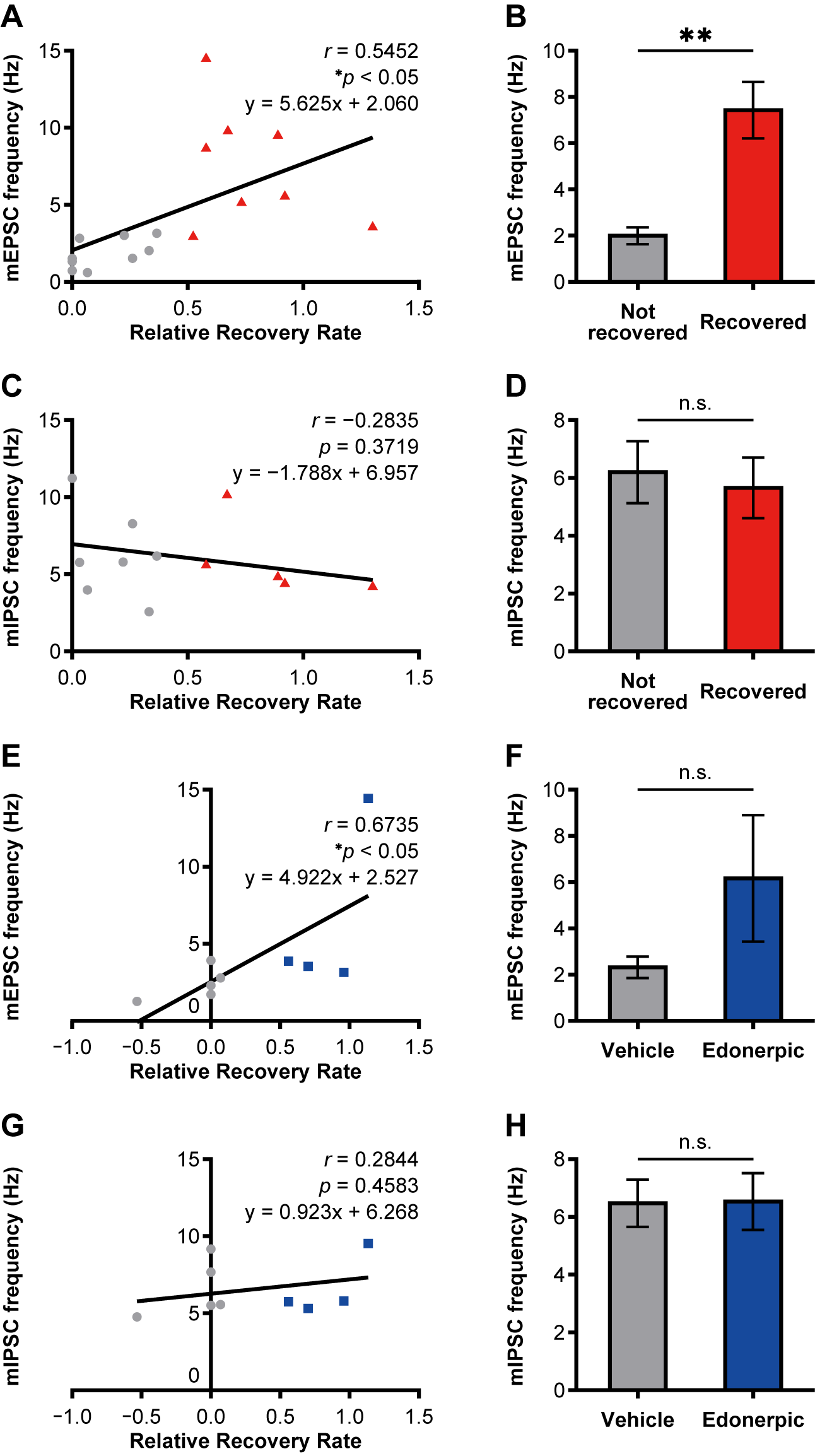

Supplement: SUPPLEMENTARY FIGURE 2 — The balance of spontaneous mEPSC/IPSC frequency is also disrupted in naturally recovered rats and maintained in edonerpic maleate-induced recovered rats. (A) Correlation between mEPSC frequency (Hz) and RRR in mildly injured rats. Gray circles indicate the plot of “not recovered (RRR < 0.4)” rats, and red triangles indicate the plot of “recovered (RRR ≥ 0.4)” rats (same in C). Data were analyzed by Pearson correlation coefficient. (B) Average frequencies of spontaneous mEPSCs. Comparison of not recovered (n = 9 animals, 11 cells) and recovered (n = 8 animals, 14 cells) rats. Unpaired t-test (**p < 0.01). (C) Correlation between mIPSC frequency (Hz) and RRR in mildly injured rats. Data were analyzed by Pearson correlation coefficient. (D) Average frequencies of spontaneous mIPSCs. Comparison of not recovered (n = 7 animals, seven cells) and recovered (n = 5 animals, eight cells) rats. Unpaired t-test. n.s., not significant. (E) Correlation between mEPSC frequency (Hz) and RRR in severely injured rats. Gray circles indicate the plot of “vehicle treated control (RRR < 0.4)” rats, and blue squares indicate the plot of “edonerpic maleate-induced recovered (RRR ≥ 0.4)” rats (same in G). Data were analyzed by Pearson correlation coefficient. (F) Average frequencies of spontaneous mEPSCs. Comparison of vehicle treated control (n = 5 animals, 12 cells) and edonerpic maleate-induced recovered (n = 4 animals, nine cells) rats. Unpaired t-test. n.s., not significant. (G) Correlation between mIPSC frequency (Hz) and RRR in severely injured rats. Data were analyzed by Pearson correlation coefficient. (H) Average frequencies of spontaneous mIPSCs. Comparison of control (n = 5 animals, 12 cells) and edonerpic maleate-induced recovered (n = 4 animals, nine cells) rats. Unpaired t-test. n.s., not significant. [file Image_2.TIF]

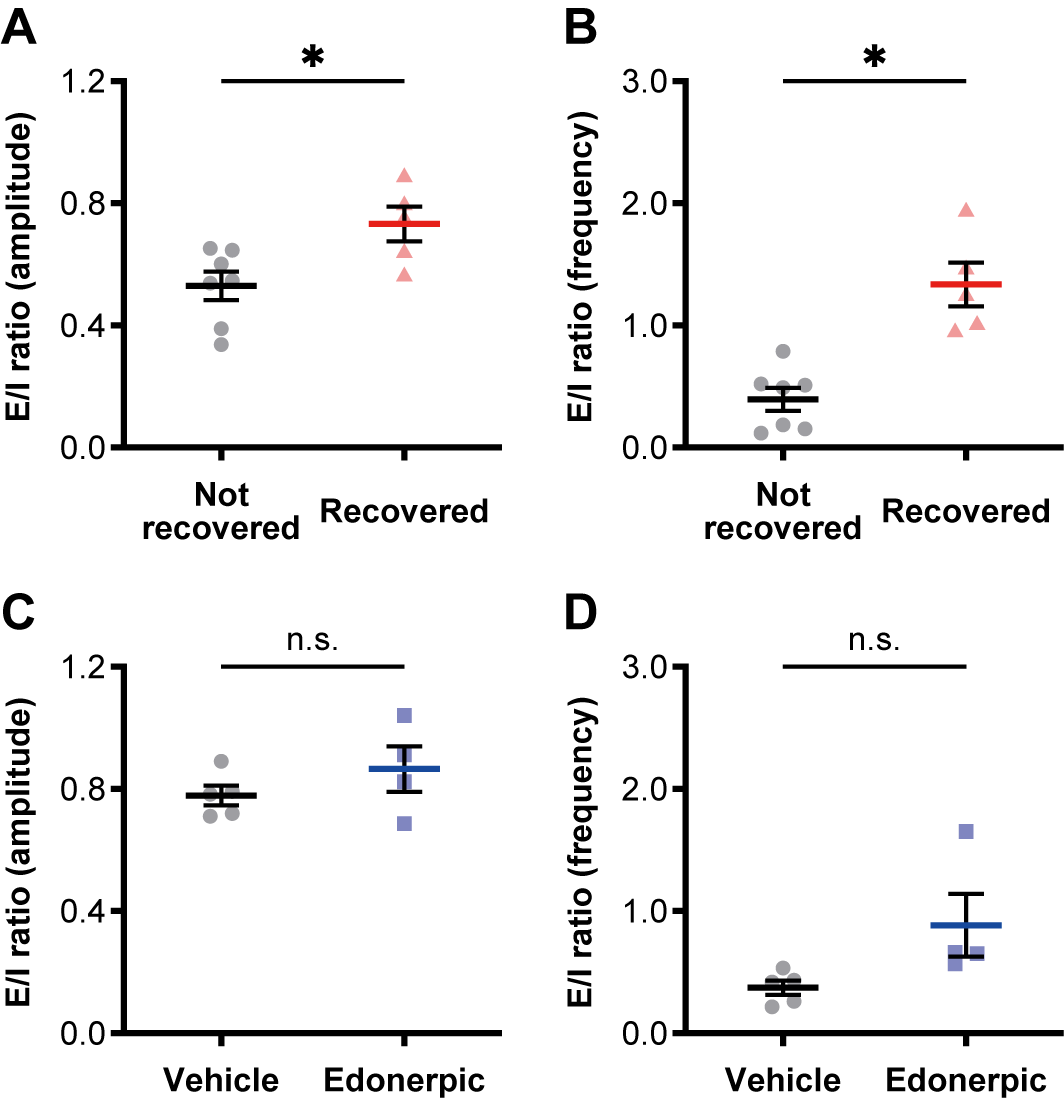

Supplement: SUPPLEMENTARY FIGURE 3 — Disrupted E/I ratio in naturally recovered rats, but not in edonerpic maleate-induced recovered rats. (A) E/I ratio of post-synaptic current amplitude in mildly injured rats. Comparison of not recovered (n = 7 animals, seven cells) and recovered (n = 5 animals, eight cells) rats. Unpaired t-test (*p < 0.05). (B) E/I ratio of post-synaptic current frequency in mildly injured rats. Comparison of not recovered (n = 7 animals, seven cells) and recovered (n = 5 animals, eight cells) rats. Unpaired t-test (*p < 0.05). (C) E/I ratio of post-synaptic current amplitude in severely injured rats. Comparison of vehicle treated control (n = 5 animals, 12 cells) and edonerpic maleate-induced recovered (n = 4 animals, nine cells) rats. Unpaired t-test. n.s., not significant. (D) E/I ratio of post-synaptic current frequency in severely injured rats. Comparison of vehicle treated control (n = 5 animals, 12 cells) and edonerpic maleate-induced recovered (n = 4 animals, nine cells) rats. Unpaired t-test. n.s., not significant. [file Image_3.TIF]
